# An ecological genetic delineation of local seed-source provenance for ecological restoration

**DOI:** 10.1002/ece3.595

**Published:** 2013-06-05

**Authors:** Siegfried L Krauss, Elizabeth A Sinclair, John D Bussell, Richard J Hobbs

**Affiliations:** 1 Botanic Gardens and Parks AuthorityFraser Avenue, West Perth, Western Australia, 6005, Australia; 2 School of Plant Biology, University of Western AustraliaNedlands, Western Australia, 6009, Australia; 3 ARC CoE Plant Energy Biology, University of Western AustraliaNedlands, Western Australia, 6009, Australia

**Keywords:** AMOVA, AFLP, ANOSIM, BayeScan, *Banksia*, Proteaceae

## Abstract

An increasingly important practical application of the analysis of spatial genetic structure within plant species is to help define the extent of local provenance seed collection zones that minimize negative impacts in ecological restoration programs. Here, we derive seed sourcing guidelines from a novel range-wide assessment of spatial genetic structure of 24 populations of *Banksia menziesii* (Proteaceae), a widely distributed Western Australian tree of significance in local ecological restoration programs. An analysis of molecular variance (AMOVA) of 100 amplified fragment length polymorphism (AFLP) markers revealed significant genetic differentiation among populations (Φ_PT_ = 0.18). Pairwise population genetic dissimilarity was correlated with geographic distance, but not environmental distance derived from 15 climate variables, suggesting overall neutrality of these markers with regard to these climate variables. Nevertheless, Bayesian outlier analysis identified four markers potentially under selection, although these were not correlated with the climate variables. We calculated a global *R*-statistic using analysis of similarities (ANOSIM) to test the statistical significance of population differentiation and to infer a threshold seed collection zone distance of ∼60 km (all markers) and 100 km (outlier markers) when genetic distance was regressed against geographic distance. Population pairs separated by >60 km were, on average, twice as likely to be significantly genetically differentiated than population pairs separated by <60 km, suggesting that habitat-matched sites within a 30-km radius around a restoration site genetically defines a local provenance seed collection zone for *B. menziesii*. Our approach is a novel probability-based practical solution for the delineation of a local seed collection zone to minimize negative genetic impacts in ecological restoration.

## Introduction

Ecological restoration – the process of assisting the recovery of an ecosystem that has been degraded, damaged, or destroyed – is a rapidly emerging scientific discipline (Clewell and Aronson 2007). Decisions on the sourcing of vast quantities of germplasm (typically seed) should be underpinned by fundamental ecological and evolutionary principles that drive spatial genetic structure within species (Hufford and Mazer 2003; McKay et al. 2005). Consideration of the potentially negative consequences of introducing non-local provenance genotypes has largely focused on maladaptation, whereby non-local genotypes are considered to have a fitness disadvantage over local genotypes, and their use can lead to restoration failure or inefficiencies (McKay et al. 2005; Bischoff et al. 2010). Other genetic issues underpinning a concern with sourcing local, rather than composite, provenance genotypes for restoration include outbreeding depression (Hufford and Mazer 2003; Edmands 2007; Goto et al. 2011; Hufford et al. 2012), genetic swamping (Potts et al. 2003), and an erosion of spatial genetic structure leading to negative consequences for the conservation of within-species biodiversity (Krauss and He 2006). In addition, it is now recognized that heritable genetic variation among populations within especially keystone or dominant species can, through the extended phenotype, affect entire communities and ecosystems (Whitham et al. 2006; Lankau and Strauss 2007; Barbour et al. 2009), so the introduction of non-local provenance genotypes can rapidly alter local ecosystem diversity, as well as erode resistance to biological (weed) invasion (Saltonstall 2002). Thus, the maintenance of landscape genetic structure within a species may also be essential for maintenance of a diversity of interacting/competitor species, furthering the goals of ecological restoration by fostering species interactions (e.g., vital plant/pollinator interactions) and ultimately functioning and resilient biological systems (Crutsinger et al. 2006; Menz et al. 2011; Ritchie and Krauss 2012).

Seed sourcing for ecological restoration, however, is a highly complex issue, and there are situations where non-local provenance sourcing is warranted (Broadhurst et al. 2008; Sgro et al. 2011; Byrne et al. 2011; Weeks et al. 2011). Key issues here include composite provenancing to maximize evolutionary potential and avoid potentially inbred seed from small fragmented source populations (Broadhurst et al. 2008), restoring landscapes that have been highly altered from their natural state (Lesica and Allendorf 1999), and addressing climate change scenarios (Sgro et al. 2011). In addition, restoration targets differ depending on such aspects of landscape matrix (intact or fragmented), degree of disturbance (e.g., increased salinity or vegetation clearing), and available funding (Krauss in press).

However, it is not our intention to address here the relative merits of different seed sourcing strategies. Rather, we are concerned with the situation when the ecological restoration of highly diverse, functional and integrated plant communities that reflect pre-disturbance communities is a specific objective, and the landscape matrix for seed sourcing is relatively intact. Under this scenario, the sourcing of genetically diverse seed from within the local provenance, or from a defined local seed collection zone, is generally recognized as desirable (McKay et al. 2005). In the pursuit of this objective, a key question becomes, how local is local? Practical guidelines that delineate seed collection zones or regions of provenance for species or regions have been developed (e.g., Mortlock 2000; Forestry Commission Scotland 2006). However, these general guidelines are often best guesses based on biological, ecological or climatic criteria that may not accurately reflect genetic structure, and therefore can be in error, even overly restrictive, for individual species. The application of molecular markers for an assessment of population genetic structure can make a vital contribution to this objective, and this is an increasingly important practical application of ecological genetics (Bussell et al. 2006). Despite a demand from restoration practitioners, detailed applied genetic information on the extent of local genetic provenance is not known for all but a few species.

Molecular markers can be used to analyze population genetic variation and – in addition to other known information on for example morphology, taxonomy, polyploidy, and habitat – can make a useful contribution to the delineation of local genetic provenance for a species. A practical contribution from population genetics requires efficient sampling by a molecular tool that detects sufficient diversity and is rapidly applied to new species (for which typically little is known about the genome). Ideally the markers should capture the main sources of neutral and non-neutral population genetic variation – local adaptation, genetic drift, gene flow, and mutation – and the interactions between these sources. Given the complexity of these genetic objectives, there is no ideal single molecular marker technique. However, the polymerase chain reaction (PCR)-based multi-locus DNA fingerprinting technique amplified fragment length polymorphism (AFLP) is perhaps the most suitable marker to meet these objectives. AFLP efficiently delivers information for many markers from across the genome, and typically at least some of these markers detect signatures of natural selection and can strongly influence the overall patterns of genetic differentiation detected (Beaumont and Balding 2004; Foll and Gaggioti 2008; Stingemore and Krauss 2013). New research on the human genome has also reinforced that “intergenic”, or non-protein coding, DNA plays a crucial role in gene regulation (ENCODE project consortium 2012), which highlights the potential of markers that have been typically considered “neutral”, such as AFLP, to illustrate genetic diversity that is of evolutionary significance.

Here, we seek to delimit local provenance seed-source zones in an iconic Western Australian species, *Banksia menziesii* R.Br (firewood banksia). *Banksia menziesii* is a key species used in the ecological restoration of disturbed and degraded sites across the Swan Coastal Plain (SCP), a region of high species diversity within the South-West Australian Floristic Region (SWAFR), an international biodiversity hotspot (Myers et al. 2000; Hopper and Gioia 2004; Rokich and Dixon 2007). With the key objective of making a novel practical contribution toward genetic guidelines for seed sourcing to maximize ecological restoration success, we generate and assess AFLP data for *B. menziesii* populations across the entire range of the species to (i) estimate population genetic diversity and differentiation parameters, (ii) provide genetic guidelines for local provenance seed sourcing for the establishment of ecologically restored populations that have high evolutionary potential and are genetically integrated with existing local populations, and (iii) more generally, define a threshold distance that has biological significance, beyond which there are potentially negative restoration consequences from collecting seed for restoration. Additionally, we employ a population genomics approach to look for signatures of natural selection in the markers we have employed, through the identification of molecular marker outliers as well as associations between markers and environmental data obtained for each site.

## Materials and Methods

### Species and sampling sites

**Figure 1:**
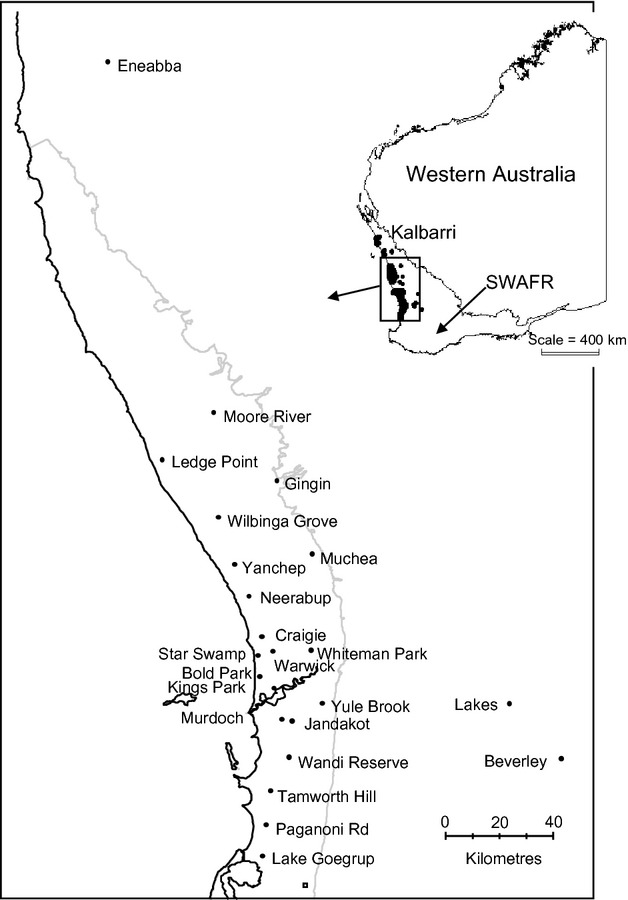
Map showing the distribution of *Banksia menziesii* in south-west Australia, and the location of 24 populations sampled for genetic analysis. Note all locations except Kalbarri, Eneabba, Beverley, and Lakes occur on the Swan Coastal Plain (SCP), a narrow sandplain bounded by the coastline to the west and the Darling Scarp to the east.

*Banksia menziesii* is a dominant over-story species, widespread along the deep sandy soils of the SCP (Fig. 1) (Taylor and Hopper 1988). The eastern limit of the distribution is restricted by the heavy soils of the Darling Scarp, although some isolated populations occur inland on sand lenses. In the northern part of its range, plants are usually lignotuberous shrubs up to 3 m tall, while to the south plants typically grow into small trees, 3–10 m in height (Taylor and Hopper 1988). Populations are typically larger than 100 individuals, with an estimated 77% of trees being outside of conservation reserves (Taylor and Hopper 1988). Flowering occurs between February and August (George 1987), with flower color variation ranging from dark red to pink and yellow (Fuss and Sedgley 1990).

Reproductive traits include obligate outcrossing (Scott 1980), limited time of pollen viability (<24 h, Ramsey and Vaughton 1991), extremely low seed set (Whelan and Burbidge 1980), and low recruitment rates for seedlings (Cowling and Lamont 1987). These traits when combined with susceptibility to “die-back” *Phytophthora cinamomi* (McCredie et al. 1985; Shearer and Dillon 1996), altered fire regimes (Hobbs and Atkins 1990), and reduced availability of ground water (Groom et al. 2001; Zencich et al. 2002) all combine to contribute to a decline in vigor of *B. menziesii* populations. Differences in growth form (Taylor and Hopper 1988) and reproductive traits have been reported between northern and southern populations, as well as clinal variation in serotiny (Lamont et al. 1994). Habitat fragmentation due to urban development has been significant within the southern part of the range (greater Perth Metropolitan area), while a possible decline in the northern part of the range (Eneabba sand plain, Cowling and Lamont 1987) may be due to increasing aridity and more frequent fires (Lamont et al. 1994).

Fresh leaf material was collected from mature plants in 24 natural and largely undisturbed locations across the entire species range, including naturally fragmented inland sand lens populations (Beverley and Lakes; Fig. 1, Table 1). Leaves were collected from plants >10 m apart to avoid sampling close relatives within each location. The precise location of each plant was recorded using global positioning system (GPS) (AGD84). Sample sites occurred within distinct geomorphological zones running north-south – Coastal Quindalup, Spearwood and Bassendean dunes on the SCP, and naturally fragmented sand lens populations north of the SCP and within the Darling Range east of the SCP.

**Table 1:** *Banksia menziesii* sampling locations, sample size (*N*), proportion of 100 AFLP markers scored that were polymorphic (PPL), Shannons information index (*I*), and annual rainfall

Population	Latitude (S)	Longitude (E)	*N*	PPL	*I*	Annual rainfall (mm)
*Kalbarri*	−28.09	114.33	10	0.23	0.12	443.7
*Eneabba*	−29.83	115.26	9	0.22	0.12	523.3
Moore River	−31.03	115.62	16	0.31	0.15	618.8
Ledge Point	−31.19	115.44	14	0.36	0.16	652.2
Gingin	−31.26	115.83	18	0.34	0.15	639.1
Wilbinga Grove	−31.39	115.63	14	0.37	0.18	690.5
Muchea	−31.51	115.95	16	0.25	0.13	707.3
Yanchep	−31.55	115.69	14	0.42	0.19	710.6
Neerabup	−31.66	115.73	24	0.48	0.21	747.5
Craigie	−31.79	115.78	16	0.38	0.19	757.8
Whiteman Park	−31.84	115.94	17	0.28	0.13	752.3
Warwick	−31.84	115.81	12	0.28	0.14	766.1
Star Swamp	−31.86	115.76	15	0.33	0.14	743.4
Bold Park	−31.93	115.77	20	0.39	0.18	754.4
Kings Park	−31.97	115.82	20	0.38	0.17	774.9
Yule Brook	−32.02	115.98	19	0.33	0.15	797.2
*Lakes*	−32.02	116.61	11	0.30	0.15	544.0
Murdoch	−32.07	115.84	9	0.30	0.16	788.7
Jandakot	−32.08	115.88	13	0.33	0.15	805.7
Wandi Reserve	−32.20	115.87	12	0.32	0.16	813.9
*Beverley*	−32.21	116.78	16	0.33	0.15	446.0
Tamworth Hill	−32.32	115.81	18	0.31	0.14	797.2
Paganoni Rd	−32.44	115.79	14	0.32	0.16	824.8
Lake Goegrup	−32.54	115.78	12	0.36	0.18	830.9
Mean per population			15.0	0.33	0.16	
Overall			359	0.79		

Italicized populations indicate climatic outliers.

### DNA extraction and AFLP profiling

Genomic DNA was extracted from freshly collected material using a modified CTAB protocol (Carlson et al. 1991; He et al. 2004), with all plant material ground in liquid nitrogen prior to extraction. AFLP (Vos et al. 1995; Mueller and Wolfenbarger 1999) DNA fingerprints were generated for each sample using fluorescently labeled primers (primer combinations were mCTA/eACT, mCTA/eAGG, mCAG/eACC) following Krauss (1999). Bands were visualized using an ABI 377 sequencer and Genescan software (Applied Biosystems, Foster City, CA) with internal size standard (ROX, Applied Biosystems). The presence (1) or absence (0) of amplified fragments between 85 and 458 base pairs was scored unambiguously with the aid of Genotyper software (Applied Biosystems). Reproducibility and consistency of the markers scored was confirmed by duplicate runs for selected samples and the inclusion of standards on all gels.

### Data analyses

Genetic diversity was measured as the proportion of all markers that were polymorphic (PPL) and Shannon's diversity index (*I*) within populations, and overall, using GenAlEx v6.5b3 (Peakall and Smouse 2012). The partitioning of the total genetic variation into within and among population components was assessed by an analysis of molecular variance (AMOVA) using GenAlEx v6.5b3. Non-metric multidimensional scaling (MDS) was used to visualize the relative degree of genetic dissimilarity among all populations from a Euclidean distance metric, using Primer v6 (Clarke and Gorley 2006). Clusters of populations were inferred from unweighted pair group method with arithmetic mean analyses and represented on ordinations as ellipses, using Primer v6.

An analysis of pairwise population similarities was used to test for significant differences between all pairs of populations, using the ANOSIM (analysis of similarities) function in Primer V6 (Clarke and Gorley 2006). ANOSIM generates a nonparametric test statistic, *R*, based on the ranked similarities among all pairs of samples within populations compared to that of all pairs of samples among populations, with significance assessed by permutation testing (5000 permutations). Significance of pairwise population differentiation was also determined by permutation testing, and by comparison to the global *R*-statistic (Clarke and Gorley 2006). *R* values usually range from 0 (no difference between populations as pairwise similarities between and within sites are the same on average) to 1 (all pairwise similarities between populations are larger than those within populations) (Chapman and Underwood 1999). We assessed the relationship between genetic distance (assessed by pairwise *R*) and linear geographic distance visually, and by a Mantel test (with 9999 iterations) in Genalex v6.5b3. Sampling locations were plotted using GPS points on OziExplorer (http://www.oziexplorer.com) to generate accurate geographic distances (km) between each pair of locations.

Climate data (Annual, January and June means for each of potential evapotranspiration, solar radiation, rainfall, maximum temperature and minimum temperature) were obtained from the Australian Bureau of Rural Sciences Natural Resources Data Library. These data were then extrapolated to sample population coordinates with Diva geographic information system (http://www.diva-gis.org/).

Relationships among the genetic, geographic and climate distance matrices were assessed by simple and partial Mantel tests using MantelTester (http://manteltester.berlios.de/), which uses the Zt software tool (Bonnet & Van de Peer 2002). Partial Mantel tests enable a test of the correlation between two distance matrices while controlling for the effect of a third, in order to remove spurious correlations. The pairwise *R* was used as a measure of genetic distance, geographic distance was calculated from GPS coordinates using GenAlEx v6.5b3, and climate distance was calculated as Euclidean distance from climate variables that were each first standardized for equal weighting, in Primer V6. Simple Mantel tests assessed the null hypothesis that distances in each pair of matrices are independent. Partial Mantel tests assessed the correlation between matrix A and B while controlling the effect of the third matrix C, in order to remove spurious correlations, through permutations (here 10,000) of the residuals of a null model (Anderson & Legendre 1999).

To detect signatures of natural selection on individual genetic markers, a Bayesian approach generalized from the method of Beaumont and Balding (2004) was implemented within the program BayeScan V.2.1 (Foll and Gaggioti 2008; Foll et al. 2010) to allow direct estimation of the posterior probability that a given locus is under selection. The rationale was to discriminate between the effects on the partitioning of population genetic variation (*F*_ST_ values) that are specific to each population and to each locus. The method uses a hierarchical Bayesian approach to estimate the posterior probabilities of two alternative models, one including the effects of selection and one excluding it. The results are expressed as posterior odds (PO), which indicate for each locus the ratio of posterior probability of the selection model against the neutral model and interpreted as different levels of evidence of selection according to Jeffreys scale. We used a threshold of PO >100 (“decisive”) for a marker to be considered under selection. This corresponds to a posterior probability of >0.99 for the model accounting for selection. BayeScan was run with 20 pilot runs with a burn-in of 50,000 followed by 50,000 iterations each, a sample size of 5000, a thinning interval of 10, and an *F*_IS_ set at a mean of 0.05 based on known data for complete outcrossing in *B. menziesii* (Scott 1980). In addition, we used the false discovery rate (FDR) to control for multiple testing. The FDR is defined as the expected proportion of false positives among outlier markers. In this context, BayeScan defines a *q*-value, which is the minimum FDR at which a locus may become significant. A *q*-value of 1% (−log_10_(*q*) = −2) means that 1% of corresponding outlier markers (those having a *q*-value <1%) are expected to be false positives. We then conducted simple and partial Mantel tests, as outlined above, on individual markers identified by the Bayesian allele frequency test.

## Results

In total, 100 AFLP markers were scored for 359 *B. menziesii* plants sampled from 24 locations across the geographic range of the species, of which 79 were polymorphic (Table 1). The PPL within populations ranged between 0.22 and 0.48 (mean = 0.33), and the Shannon diversity index (*I*) ranged between 0.12 and 0.21 (mean = 0.16). AMOVA partitioned 82% of the total variation among individuals within populations (Table 2), and 18% among populations (Φ_PT_ = 0.18), which was significantly different from zero (*P* < 0.01; Table 2).

**Table 2:** Summary AMOVA table for 24 populations of *Banksia menziesii* genotyped with 100 polymorphic markers

Source	df	SS	MS	Estimated variance	% Total variation
Among Populations	23	550.3	23.930	1.186	18
Within Populations	354	1888.8	5.330	5.336	82
Total	377	2439.1		6.522	100

**Figure 2:**
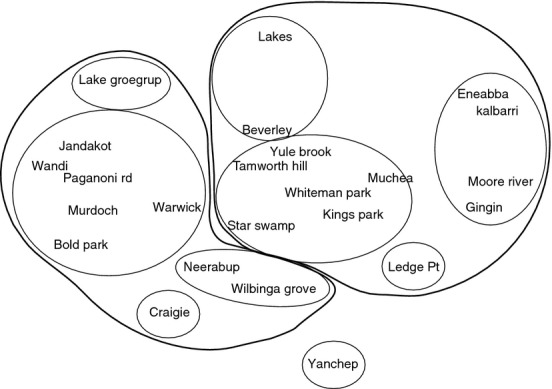
Multi-Dimensional Scaling (MDS) plot of genetic dissimilarity from 79 polymorphic AFLP markers for *Banksia menziesii* plants from 24 locations. Confidence ellipses are drawn from clusters identified from unweighted pair group method with arithmetic mean (UPGMA) analysis.

MDS ordination of the genetic data showed an arrangement of populations that was largely associated with geographic proximity (Fig. 2). A notable exception though was Bold Park and Kings Park, which were relatively genetically differentiated despite a geographic distance of 8 km. An overall association between average pairwise population genetic dissimilarity with pairwise population geographic distance was also reflected in a significant Mantel test (*R*^2^ = 0.33; *P* < 0.01).

**Figure 3:**
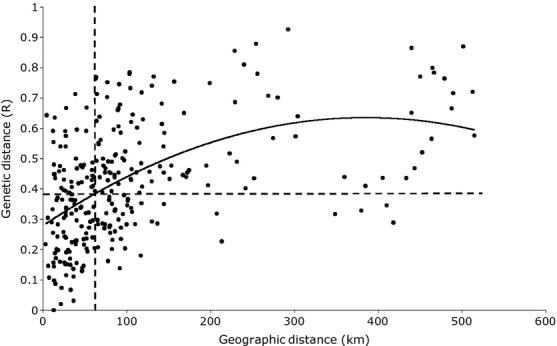
Plot showing pairwise population *R* values representing genetic distance for 24 populations from 79 polymorphic AFLP markers against geographic distance for population pairs of *Banskia menziesii*, with a polynomial line of best fit. The threshold significance (*R* = 0.38, horizontal dashed line) equates to a geographic distance of ca. 60 km (vertical dashed line). Above 60 km, two thirds of all pairwise *R* values fall above the global threshold, while below 60 km two thirds of all pairwise *R* values fall below the global threshold.

ANOSIM for all markers generated a global *R* of 0.39, which was significantly different from 0 (*P* < 0.001). Approximately half (149/276) of all pairwise population tests were significant at *P* < 0.001 as determined by 999 random permutations. There was a significant positive correlation between genetic distance (as measured by *R*) and geographic distance (Mantel test *R*^2^ = 0.24, *P* < 0.01). The global *R* of 0.39 intersected the line of best fit through these points that corresponded to a geographic distance of ca. 60 km (Fig. 3). For the distance class 0–60 km, there were 118 pairwise populations, of which 78 (66%) generated an *R* < 0.39, and 40 (34%) generated an *R* > 0.39. This result was similar when calculated for the 0–20 km and 0–30 km distance classes. Beyond 60 km, there were 158 pairwise population comparisons, of which 49 (31%) generated an *R* < 0.39, and 109 (69%) generated an *R* > 0.39. Beyond 150 km, these values were 12% and 88%, respectively.

Simple Mantel tests showed significant relationships between all pairs of distance matrices (Table 3). Partial Mantel tests showed significant relationships between geographic and climate distance when controlled for genetic distance and genetic and geographic distance when controlled for climate distance. However, the relationship between genetic and climate distance, when controlled for geographic distance, was not significant (Table 3). This result indicated that genetic distance was significantly correlated with geographic distance independently of climate distance.

**Table 3:** Simple (lower diagonal) and partial (upper diagonal) Mantel test results for geographic distance (GEO), climate distance (CLI), and genetic distance (GEN) for 100 AFLP markers scored for 24 populations of *Banksia menziesii*

All	GEO	CLI	GEN
GEO		0.790**	0.466**
CLI	0.740**		0.419**
	Controlled for GEN		
GEN	0.243*	0.093 (NS)	
	Controlled for CLI	Controlled for GEO	

** *P* < 0.001;

* *P* < 0.01; NS, nonsignificant (*P* > 0.05). Probability values are Bonferroni adjusted to account for multiple comparisons.

**Figure 4:**
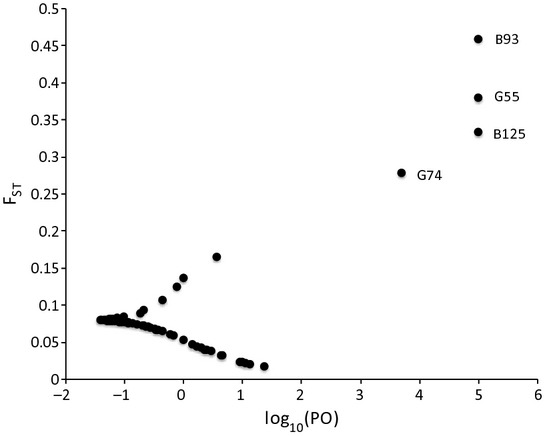
BayeScan 2.1 plot of the global genome scan for 79 polymorphic AFLP markers from 359 individuals and 24 populations of *Banksia menziesii*. *F*_ST_ is plotted against log10 of the posterior odds (PO), which identifies four outlier markers, which are candidates for being under positive selection.

Four markers were identified as significant outliers by BayeScan analysis, with *F*_ST_ values that were 2–3 times those of non-outliers, with strongly positive alpha coefficients (1.5–2.3), “decisive” posterior probabilities (log_10_PO >2), and a FDR of <0.01% (log_10_*q* <−4.0) (Fig. 4). In addition, marker -specific Φ_PT_ values from AMOVA for these four markers were 0.40–0.61, compared to overall Φ_PT_ = 0.18. Therefore, these markers are likely candidates subject to divergent selection (Fischer et al. 2011). Partial Mantel tests showed no significant association with climate distance nor geographic distance, when controlled for geographic distance and climate distance, respectively, for each of these four outlier markers. Two pairs of markers showed significant associations (*P* < 0.001), one positive (G55 and B93) and one negative (G74 and B93). An MDS ordination of genetic dissimilarity from these four markers (not shown) largely reflected that generated from all markers (Fig. 2), which was supported by a significant Mantel test (*R*^2^ = 0.52; *P* = 0.01) between these two genetic dissimilarity matrices.

ANOSIM for the four outlier markers generated a global *R* of 0.45. Plotting (not shown) genetic distance (as measured by *R* based on these four markers) against geographic distance resulted in a weaker positive correlation (Mantel test *R*^2^ = 0.08, *P* < 0.01) than for all markers combined (Fig. 3), and the global *R* of 0.45 on this plot corresponded to a distance of ca. 100 km.

**Figure 5:**
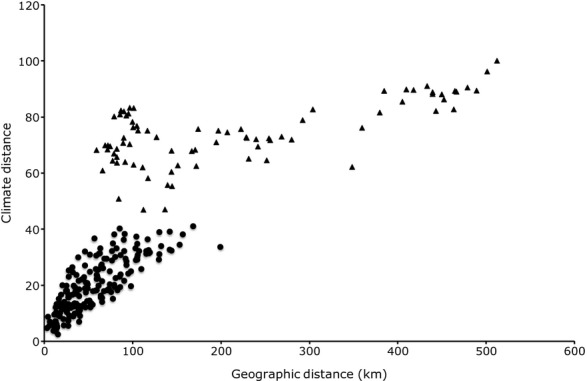
Plot showing pairwise site climate distance (based on 15 climate variables) against geographic distance for 24 sites from which *Banksia menziesii* was sampled for genotyping. Circles indicate comparisons between sites on the Swan Coastal Plain (SCP), triangles indicate comparisons between SCP populations and those off the SCP (i.e., Kalbarri, Eneabba, Beverley and Lakes).

**Figure 6:**
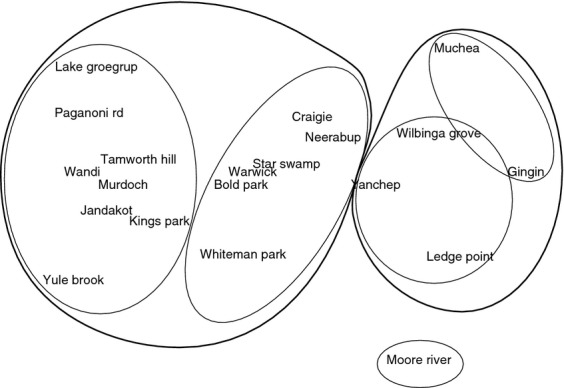
Multidimensional Scaling (MDS) plot of 15 climate variables for 20 Swan Coastal Plain locations, with confidence ellipses drawn from clusters identified from unweighted pair group method with arithmetic mean (UPGMA) cluster analysis. Note that the four geographic outlier sites (Kalbarri, Eneabba, Beverley and Lakes) are not included on this plot due to the relative magnitude of climatic dissimilarity (3–4× that within the SCP sites) between these locations and the SCP locations. This plot provides a guide to habitat matching among sites on the basis of climate. Climate variables were annual, January and July averages for each of mean rainfall, potential evapotranspiration, solar radiation, maximum and minimum temperatures and were obtained from the Australian Bureau of Rural Sciences Natural Resources Data Library and extrapolated to location coordinates with Diva geographic information system (GIS).

A marked disjunction in climate dissimilarity (climate distance >45) was found between the SCP populations and non-SCP populations (Kalbarri, Eneabba, Lakes, Beverley) (Fig. 5). While climate distance was significantly associated with geographic distance for the SCP sites, there was significant clustering of sites into far north (Moore River), north (centered around Wilbinga Grove) and south (centered around the Swan River) (Fig. 6). The southern cluster was further clustered into sites north and south of the Swan River (Fig. 6). Of significance was the clustering of Kings Park with sites south of the Swan River, despite being located north of the Swan River and only 8 km inland from the Bold Park site (Fig. 1).

## Discussion

There is a rapidly increasing demand for the seed of native plants for ecological restoration activities worldwide. With this is an urgent requirement for improved practitioner guidelines on seed sourcing for better restoration outcomes that minimize potentially negative genetic consequences on ecological restoration and the restoration of diverse, functional and integrated biological systems (Mortlock 2000; Falk et al. 2001; Ying and Yanchuk 2006; Broadhurst et al. 2008; Bischoff et al. 2010; Mijnsbrugge et al. 2010). We have addressed this need by utilizing a nonparametric multivariate approach (ANOSIM) that is relatively new in population genetics, but widely used in ecology (Clarke and Gorley 2006) and well suited for dominant marker data, to define the scale of local provenance from population genetic data for *B. menziesii*. In particular, we used the relationship between the *R*-statistic (globally and for all pairwise population comparisons as a measure of genetic differentiation) and geographic distance, to infer a biologically significant threshold seed collection distance. Beyond this threshold distance we suggest that there are potentially negative consequences from collecting seed for ecological restoration, although this ultimately requires experimental confirmation. This objective materially enhances the generic seed collecting guideline extremes of composite provenancing (Broadhurst et al. 2008; Sgro et al. 2011; Weeks et al. 2011) or, in contrast, of collecting seed as locally as possible (McKay et al. 2005; Bischoff et al. 2010).

For 79 variable AFLP markers in *B. menziesii*, we have attached significance to a global *R* of 0.39 (rather than *R* = 0.45 for the four outlier markers, for reasons we discuss below), which corresponded to a geographic distance of 60 km, or equivalently a 30-km radius around a restoration site. The unique strength of this analysis is that it enabled us to assign probabilities of genetically matching pairs of populations at various distance classes, depending on the proportion of points falling below and above the global *R*. Within 60 km, 66% of all population pairs fell below the global *R*, and 34% above. Beyond 60 km, 31% of all population pairs fell below the global *R*, and 69% above. Consequently, the probability of genetically matching population pairs (in a relative sense compared to global *R*) by chance when separated by >60 km is half that for populations separated by <60 km. Importantly, the proportion under 60 km does not change for the smaller distance classes of 30 km, nor 20 km. In contrast, above 150 km, there is only a 1 in 10 chance of genetically matching population pairs.

However, even within 60 km, there was a large range in pairwise population *R* values, from ∼0 to almost 0.8. For example, Bold Park is a 437 Ha remnant of coastal bushland in the western suburbs of metropolitan Perth that was declared an A-class reserve in 1998 and has been the subject of the most extensive ecological restoration effort in the Perth metropolitan area (BGPA 2000). Geographically, Kings Park is one of the closest (at 8 km), and largest (346 Ha), reserves to Bold Park and the most likely source of external seed of *B. menziesii* for restoration. For these populations, *R* = 0.65. The relatively weak genetic similarity between populations in Bold Park and Kings Park has been observed in many other species (e.g., Krauss and He 2006; Sinclair and Hobbs 2009; Sinclair et al. 2010), and is likely to be driven by substrate and climatic differences. Indeed, the ordination of climate data shows this well, where Bold Park clusters with populations to the north, and Kings Park clusters with populations to the south. However, the overall relationship between *R* and geographic distance remains when specifically comparing Bold Park to all other sites, with *R* > 0.39 for 63% of population pairs separated by >60 km, and 42% of population pairs separated by <60 km.

This example serves to demonstrate the need to qualify the genetically determined threshold seed collection zone distance of 60 km with the recommendation that habitat and climate matching of the restoration site and potential seed-source populations within the threshold distance should be conducted wherever possible to increase the probability of matching genetically adapted seed-source populations with those in or around the restoration site (McKay et al. 2005). For example, the SCP is comprised of distinct geomorphological landforms in the form of three narrow parallel sand dune systems running north to south (Quindalup, Spearwood and Bassendean dunes), and the alluvial Pinjarra Plain (Seddon 2004), and this information should be included in seed sourcing decisions. As these geomorphological elements largely run from north to south in relatively narrow strips, constraining the east-west seed-source distance from a restoration site (to remain within the same geomorphological system) is likely to be much more important than constraining the north–south distance. This emphasis also largely tracks climate variation, where, for example, rainfall gradients are steeper running east to west than they are running north to south. However, subtle variation within these landform and climatic features, for example in the neutral and chemical composition of soils, can drive genecological variation that is difficult to predict from coarse environmental observations alone (Keighery and Keighery 2010). Additionally, the natural phylogeographic history of a population (e.g., age, bottlenecking, founding source, etc.) can often only be addressed by an analysis of genetic data (Nevill et al. in press). Thus, while consideration of nongenetic parameters is critical, it is the spatial structuring of genetic variation (both neutral and non-neutral) that is a key consideration for defining a local provenance, as the spatial structuring of genetic variation is a consequence of the key drivers of genetic variation – phylogeographic history, local adaptation and restricted gene flow. Consequently, we explicitly recognize the biological significance of both adaptively and genetically similar populations, or “epitypes” (Hufford and Mazer 2003). This recognition acknowledges that similar ecotypes may exhibit differences in the genetic architecture underlying their adaptive traits. Mixing epitypes may result in the breakdown of co-adapted gene complexes, even if those populations represent similar ecotypes (Hufford and Mazer 2003), which may severely limit sexual recruitment in restored populations (Hufford et al. 2012).

Four markers were identified as outliers, and therefore candidates for positive selection. None of these markers, however, were associated with the climate variables assessed here, and overall they were only weakly associated with geographic distance. The weaker association with geographic distance and the higher threshold distance, compared to analyses with all markers, suggests an underlying, yet undefined, complexity of selection driving variation at these markers, and that seed sourcing decisions made purely on the basis of geographic distance may have greater error for these putatively non-neutral than putatively neutral markers. This conclusion serves to emphasize the importance of habitat matching where possible within the defined threshold geographic distance based on overall marker variation. Detection of the variation at these outlier markers by chance alone seems unlikely given the attention to this issue by the BayeScan analysis through the robust FDR (Fischer et al. 2011). Ultimately though, as with all studies aiming to detect adaptively relevant molecular markers from genome scans (Holderegger et al. 2008), selection experiments such as transplant trials are required to test the causal links underpinning the observed variation. Given these observations, and the significant association between genetic distances derived from these four markers and all markers, we conclude that the results from the complete dataset give a more generally applicable and robust genetic prediction of a biologically significant provenance distance.

In conclusion, our novel consideration of the *R*-statistic threshold for significance of genetic distance of AFLP markers enables the quantification of probabilities of genetically matching populations as a function of geographic distance. This then establishes a solid quantitative framework for the testing of biological significance of the genetic marker results through the use of reciprocal transplant experiments (O'Brien et al. 2007; O'Brien and Krauss 2010; Travis and Grace 2010) within and beyond the threshold geographic distance for habitat-matched and unmatched populations, as well as cross-pollination studies at this scale to assess the mating consequences of mixing provenances (Heliyanto et al. 2006; Hufford et al. 2012). On-the-ground restoration activities provide unique opportunities in applied evolutionary ecology to assess these issues while achieving restoration outcomes.
